# The Genus *Chaetogaster* Baer, 1827 (Annelida, Clitellata) in Switzerland: A First Step toward Cataloguing Its Molecular Diversity and Description of New Species on a DNA Sequence Basis

**DOI:** 10.3390/biology13090693

**Published:** 2024-09-04

**Authors:** Régis Vivien, Michel Lafont, Colin Issartel, Benoît J. D. Ferrari, Patrick Martin

**Affiliations:** 1 Swiss Center for Applied Ecotoxicology (Ecotox Center), EPFL ENAC IIE-GE, 1015 Lausanne, Switzerland; benoit.ferrari@centreecotox.ch; 2 Univ Lyon, Université Claude Bernard Lyon 1, CNRS, ENTPE, UMR 5023 LEHNA, F-69622 Villeurbanne, France; michel.lafont@univ-lyon1.fr (M.L.); colin.issartel@univ-lyon1.fr (C.I.); 3 Royal Belgian Institute of Natural Sciences, Taxonomy and Phylogeny, 29 rue Vautier, B-1000 Brussels, Belgium; pmartin@naturalsciences.be

**Keywords:** aquatic oligochaetes, Naidinae, cryptic diversity, molecular systematics, genetic lineages, species delimitation

## Abstract

**Simple Summary:**

*Chaetogaster* is a genus of the subfamily Naidinae (Naididae). It includes a large number of species and is frequent in surface coarse sediments in streams. The aim of the present study is to establish a first inventory of lineages of *Chaetogaster* (=species) in Switzerland. We analyzed genetically 135 specimens collected at 6 sites in 4 streams of 4 cantons and preserved the anterior part of almost all specimens. We were able to delimit six lineages belonging to the nominal species *Chaetogaster diaphanus* (one lineage)*, Chaetogaster diastrophus* (three lineages)*, Chaetogaster langi* (one lineage), and *Chaetogaster setosus* (one lineage), as well as three lineages of unidentified *Chaetogaster* spp. We described the three cryptic lineages of *C. diastrophus* and two *Chaetogaster* spp. lineages as new species. The prospects of the present work are to acquire more data on the molecular diversity of this genus in Switzerland and to describe the newly found *Chaetogaster* species on a molecular/morphological basis.

**Abstract:**

The genus *Chaetogaster* belongs to the subfamily Naidinae (Naididae); it includes mostly species of small size and is diverse and abundant in surface coarse sediments in streams. The aim of the present study is to initiate an inventory of lineages (=species) of *Chaetogaster* in Switzerland. We used 135 specimens collected at 6 sites in 4 streams of 4 cantons. We sequenced the cytochrome c oxidase (COI) gene from all specimens and ITS2 and rDNA 28S from all or several specimens of each lineage that was delimited using COI data, and preserved, for morphological identifications, the anterior part of almost all sequenced specimens. We were able to delimit, based on the calculation of genetic distances and analyses of single-locus data, one lineage for *Chaetogaster diaphanus* (Gruithuisen, 1828), three within *Chaetogaster diastrophus* (Gruithuisen, 1828), one for *Chaetogaster langi* Brestcher, 1896, one for *Chaetogaster setosus* Svetlov, 1925, and three unidentified *Chaetogaster* spp. Two lineages of *Chaetogaster* spp. could correspond to a new morphological group, but this should be confirmed in more specimens. We proposed a new identification key of the nominal species and described the three *C. diastrophus* lineages and two *Chaetogaster* spp. as new species. The prospects of the present work are to complete the data of the molecular diversity of this genus in Switzerland and to describe the newly found *Chaetogaster* species on a molecular/morphological basis.

## 1. Introduction 

The genus *Chaetogaster* von Baer, 1827 belongs to the subfamily of Naidinae (Naididae) and includes species of small size (0.5–4 mm long, 0.08–0.6 mm wide) that reproduce mainly asexually by paratomy (budding and fission). While *C. limnaei* (von Baer, 1827) is an ectosymbiont and/or endoparasite on snails and other molluscs, the other *Chaetogaster* species are predators of other invertebrates and/or feed on algae [1,2]. They are frequently found during biomonitoring surveys in streams in fine to coarse sediments [3]. *Chaetogaster* species are characteristic of surface habitats and their presence in the hyporheic zone indicates infiltration of surface water [4]. In addition, they are all considered sensitive to moderately resistant to chemical pollution [4].

In total, eighteen species of *Chaetogaster* have been described based on morphological features [5]. In Switzerland, six species of *Chaetogaster* have been recorded in streams so far based on a morphological ground: *C. diaphanus* (Gruithuisen, 1828), *C. diastrophus* (Gruithuisen, 1828), *C. langi* Bretscher, 1896, *C. parvus* Pointner, 1914, *C. limnaei*, and *C. setosus* Svetlov, 1925. While *C. diaphanus*, *C. diastrophus*, and *C. langi* are frequent, *C. parvus* has been only found in some streams [3,6], *C. setosus* at two sites in one stream [7], and *C. limnaei* at one stream site [3].

Recently, Mack et al. [2] suggested that 3 commonly referenced species in North America and Europe, *C. diaphanus*, *C. diastrophus*, and *C. limnaei*, are a complex of mostly 24 cryptic species. They sequenced mitochondrial and nuclear markers of 128 *Chaetogaster* specimens from ponds, lake shores, and creeks at many locations in North America (Maryland) and Europe (mostly Scandinavian countries) and found, based on the calculation of genetic distances and analyses of single-locus and multi-locus data, 2 cryptic species for *C. diaphanus*, 3 for *C. limnaei*, and 19 for *C. diastrophus*. In other words, the overall species diversity of *Chaetogaster* has been largely underestimated on morphological grounds. For this reason, we initiated an inventory of lineages of *Chaetogaster* in Switzerland based on a DNA barcoding approach.

The aim of this study is not only to reveal the hidden diversity among *Chaetogaster* in Switzerland but also to genetically characterize the species *C. langi* and *C. setosus* and to attribute to them DNA barcode sequences. We propose an identification key to identify the nominal species *C. diaphanus*, *C. diastrophus*, *C. langi*, and *C. setosus* and describe five lineages we found within *Chaetogaster* as new species on a DNA sequence basis. 

## 2. Material and Methods

### 2.1. Specimen Collection

We used for the present study 135 *Chaetogaster* specimens collected in spring 2020–2023 and autumn 2020–2022 in surface coarse sediments at 6 different sites in 4 different streams in Canton of Vaud (Sorge River, 1 site), Canton of Bern (Suze River, 1 site), Canton of St-Gallen (Glatt River, 4 sites), and Canton Aargau (Bünz River, 1 site) as part of surveys for biomonitoring purposes (Table S1). The sediments were collected using a shovel and oligochaetes were fixed in the field with neutral buffered formalin (final concentration of formalin 10% (=formaldehyde of 4%) in the recipients). Formalin optimally fixes oligochaete specimens, and a study showed that fixation and storage of oligochaete specimens in 4% neutral buffered formalin for up to 30 days was suitable for subsequent genetic analyses [8].

In the laboratory, sediments were sieved through a column of 5 and 0.2 mm mesh size sieves. The material retained on the 0.2 mm mesh size sieve was transferred to a Tupperware box and absolute ethanol was added (final concentration of ethanol of about 100%). These boxes were preserved at −20 °C. From these samples, we selected 33 specimens of *C. diaphanus*, 67 of *C. diastrophus*, 30 of *C. langi,* and 5 of *C. setosus* using a stereomicroscope. *C. diaphanus*, *C. langi*, and *C. setosus* are recognizable using a stereomicroscope, but it is impossible to distinguish *C. diastrophus* from *C. limnaei* and *C. parvus* using the magnification of a stereomicroscope. The “*C. diastrophus*” specimens were, therefore, supposed to belong to this morphospecies (morphospecies = group of morphologically identical species) as according to the morphological inventories, it is much more frequent in Switzerland than *C. limnaei* and *C. parvus.* This was subsequently confirmed by examination with a compound microscope (see below).

Each specimen (except one specimen, *Chaetogaster* sp. MOTU 8) was cut into two pieces, the anterior part for morphological analysis and the posterior part for genetic analyses (preserved in absolute ethanol at −20 °C in Eppendorf tubes).

### 2.2. Morphological Analysis

The anterior parts were mounted on slides in a coating solution (containing lactic acid, glycerol, and Mowiol) [9] and specimens were identified to a species or morphospecies level using a compound microscope. The anterior parts (=vouchers) of all specimens, except isolates Glatt6, Nos 29, 30, 37, 38, 155, 204, 205, are deposited in the “Muséum cantonal des sciences naturelles” of Lausanne (Table S1). Almost all vouchers were photographed with an Olympus SC50 camera, with 10× and 40× magnification (photos available upon request). Out of the 135 analyzed *Chaetogaster* specimens, none were sexually mature.

### 2.3. Molecular Analyses

#### 2.3.1. DNA Extraction and Sequencing

We sequenced a fragment of 658 base pairs of the mitochondrial COI gene from 128 specimens, a fragment of 313 base pairs of the mitochondrial COI gene from 7 specimens, a fragment of 558 base pairs of the nuclear ITS2 region from 112 specimens, and a fragment of 796 base pairs of the 28S gene from 67 specimens. ITS2 and 28S were sequenced from all or several specimens per lineage delimited using COI sequences. 

Total genomic DNA was extracted using guanidine thiocyanate as described by Tkach and Pawlowski [10]. We used the LCO 1490 and HCO 2198 primers [11] for amplifying the COI fragment of 658 bp, the “mlCOIintF” and “jgHCO2198” primers [12] for amplifying the COI fragment of 313 bp, the ITS2-1 and ITS2-2 primers [13] for amplifying the ITS2 rDNA region, and the 28SC1 et 28SD2 primers [14] for amplifying the 28S region. 

PCR amplifications were performed in a total volume of 20 μL containing 0.2 μL of Taq polymerase 5 U/μL (Roche, Basel, Switzerland), 2 μL of the PCR buffer (10× concentrated) with MgCl_2_ (Roche), 0.5 μL of each primer (10 μM each), 0.4 μL of a mix containing 10 mM of each dNTP (Roche), and 1 μL of DNA template. The PCR comprised an initial denaturation step at 95 °C for 5 min, followed by 35 cycles of denaturation at 95 °C for 40 s, annealing at 44 °C for 45 s and elongation at 72 °C for 1 min, and a final elongation step at 72 °C for 8 min.

COI, ITS2, and 28S products were then bi-directionally Sanger-sequenced on an Applied Biosystems 96-capillary 3730xl instrument from Fasteris (Plan-les-Ouates, Switzerland) using the same primers (mentioned above) and following the manufacturer’s protocol. The raw sequence editing and the generation of contiguous sequences were accomplished using CodonCode Aligner (CodonCode Corporation, Centerville, MA, USA). All ITS2 and 28S sequences were obtained by direct sequencing.

The COI, ITS2, and 28S sequences are provided as Supplemental Files S1–S3 and are accessible in GenBank: COI: PP996388-PP996521, PQ198874; ITS2: PQ013378-PQ013490; 28S: PQ013491-PQ013557.

#### 2.3.2. Molecular Phylogeny

All sequences were aligned for each marker using the MUSCLE algorithm (default options) [15] implemented in Seaview v. 5.0.5 [16]. For each marker, a phylogenetic tree was inferred by maximum likelihood using IQ-TREE v. 2.2.0 for macOS [17,18], as well as optimization of its parameters, and data partitioned according to the codon position when relevant (COI: TPM3+F+G4 (codon 1), TNe+G4 (codon 2), F81+F+I (codon 3); ITS2: TNe+I+G4; 28S: TIM3+F+I+G4). Branch support was obtained with the ultrafast bootstrap with 1000 replicates [19].

#### 2.3.3. Distance Analysis

Uncorrected pairwise genetic distances in COI and ITS2 were calculated using MEGA 11 [20]. Genetic distances were calculated between sequences within consensus MOTUs (molecular operational taxonomic units) and between sequences of the different MOTUs as identified by the single-locus approaches (see below). To estimate the barcoding gap of each MOTU, we determined the maximal genetic distance within sequences of the same MOTU (=maximal intra-MOTU variation) and the minimal genetic distance between sequences of a MOTU and sequences of the other MOTUs (=minimal inter-MOTU variation). Genetic distances were also computed between the COI (*Chaetogaster*) sequences obtained by Mack et al. [2] and the COI sequences of our *Chaetogaster* MOTUs to study if our lineages were new or already found by Mack et al. [2]. For this analysis, COI sequences diverging by less than 5% of genetic divergence were considered belonging to the same lineage.

#### 2.3.4. Single-Locus Species Delimitation

Species were delineated following two complementary approaches: a distance-based method, assemble species by automatic partitioning (ASAP) [21], and a tree-based method, the “Poisson Tree Processes” (PTP) method [22]. Although very popular over the last two decades, the generalized mixed Yule-coalescent (GMYC) approach [23] was not used here because the method has been shown on several occasions to have a strong tendency to oversplit species [24,25], in particular to delimit oligochaete species [26], due to its extreme sensitivity to parametrization [27].

ASAP was run using p-distances as well as both the Jukes–Cantor (JC69) and the Kimura 2-parameter (K80) substitution models to compute the distances, in order to investigate the possible impact of different distance models on the partitioning. Analyses were performed on the dedicated public web server (https://bioinfo.mnhn.fr/abi/public/asap/, accessed on 2 May 2024).

PTP analyses were performed using multi-rate PTP (mPTP), which, unlike PTP, considers differences in intraspecific variation due to the evolutionary history or sampling of each species. mPTP is presented as an improvement of the single-rate model PTP [22], making it possible to obtain more accurate estimates than the latter [28]. PTP analyses do not require an ultrametric input tree, which is a potentially error-prone process. Therefore, the phylogenetic tree produced by the I-QTREE analysis was used as the input tree for analyses based on the PTP model. The stand-alone version of mPTP was preferred to its web implementation because certain functionalities are not available in the web service (https://mptp.h-its.org/, accessed on 2 May 2024). The last release of the pre-compiled macOS binary (mPTP 0.2.4) was downloaded from GitHub (https://github.com/Pas-Kapli/mptp, accessed on 22 August 2023).

## 3. Results

### 3.1. Identification Key of the Nominal Species

The genus *Chaetogaster* is characterized by a fusion of the first five segments forming a head containing a muscular pharynx, by the absence of ventral chaetae in segments III-V, and by the overall absence of dorsal chaetae [29]. *C. diaphanus* is different from the other species of this genus by its much larger size. *C. diastrophus* has a distinct prostomium, while *C. diaphanus*, *C. langi*, and *C. setosus* have a vestigial prostomium, or it is absent. *C. setosus* has simple pointed ventral chaetae, while *C. diaphanus*, *C. diastrophus*, and *C. langi* have bifid ventral chaetae. Based on these characters, we have created the following key to identify *C. diaphanus*, *C. diastrophus*, *C. langi*, and *C. setosus*. The mentioned sizes in the key correspond to the specimens of the present study (fixed material).

1. Body size: 1.5–4 mm long and 0.3–0.6 mm wide (at head); prostomium absent, buccal cavity large                    *C. diaphanus*

- Body size: 0.5–1.2 mm long, 0.08–0.16 mm wide (at head); prostomium present or absent; buccal cavity large or not large          2

2(1) Simple-pointed chaetae                                                    *C. setosus*

- Bifid chaetae                                                          3

3(2) Body size: 0.6–1.2 mm long, 0.11–0.16 mm wide (at head); prostomium present; buccal cavity not large to large              *C. diastrophus*

- Body size 0.5–0.7 mm long, 0.08–0.11 mm wide (at head); prostomium absent or presence of a vestigial prostomium (notch in its place at the edge of the mouth); buccal cavity generally large                                               *C. langi*

### 3.2. Delimitation of Lineages

**Figure 1:**
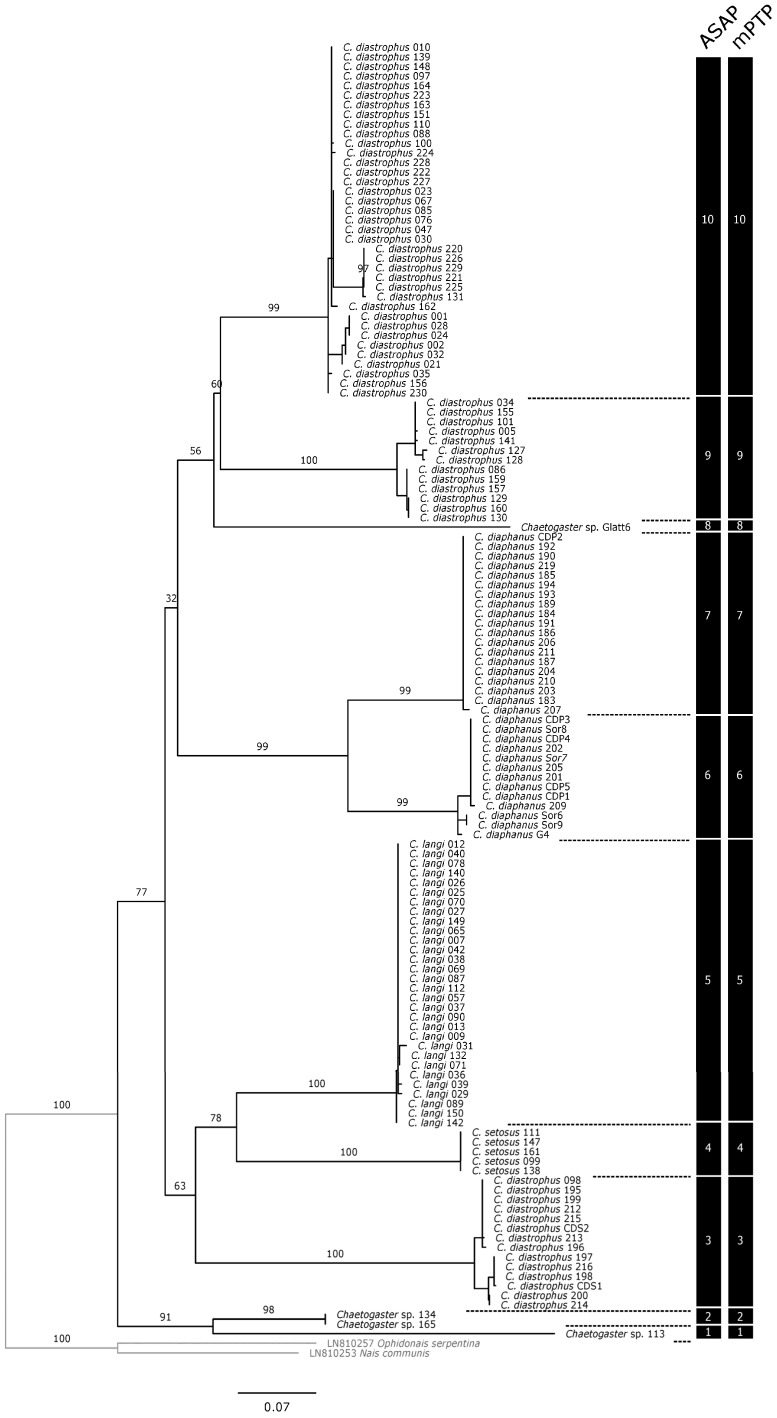
Molecular phylogeny constructed using the maximum likelihood method and COI gene fragment of *Chaetogaster* specimens. Partitions at the right side of the figure represent the results of the species delimitation analyses with single-locus methods (ASAP, mPTP). Numbers at nodes are ultrafast bootstrap values (BV). Nodes were considered supported if BVs were higher or equal to 90 [19]. For the sake of clarity, BVs are not shown within delimited MOTUs.

Species delimitation based on the COI gene leads to the identification of 10 MOTUs, either with ASAP or mPTP, which correspond to strongly supported singletons or clades in the ML tree (BV: 98–100) (Figure 1). The ASAP analyses consistently suggest the same partitioning into 10 different MOTUs, regardless of how the distances were estimated (p-distances, JC69, K80). While the grouping of specimens identified as *C. langi* and *C. setosus* remains consistent with their morphological identifications, the morphospecies *C. diaphanus* and *C. diastrophus* split into 2 (MOTU6, MOTU7) and 3 (MOTU3, MOTU9, MOTU10) groups, respectively. ASAP analyses also show a clear barcoding gap (p-distances: 4–9%; JC69: 5–9%; K2P: 4–10%). The correspondence of each lineage found in the present study to the species (=lineages) described by Mack et al. [2] is indicated in Table 1 and the mean genetic variations between lineages (present study and Mack et al. [2]) are provided in Table S2.

**Figure 2:**
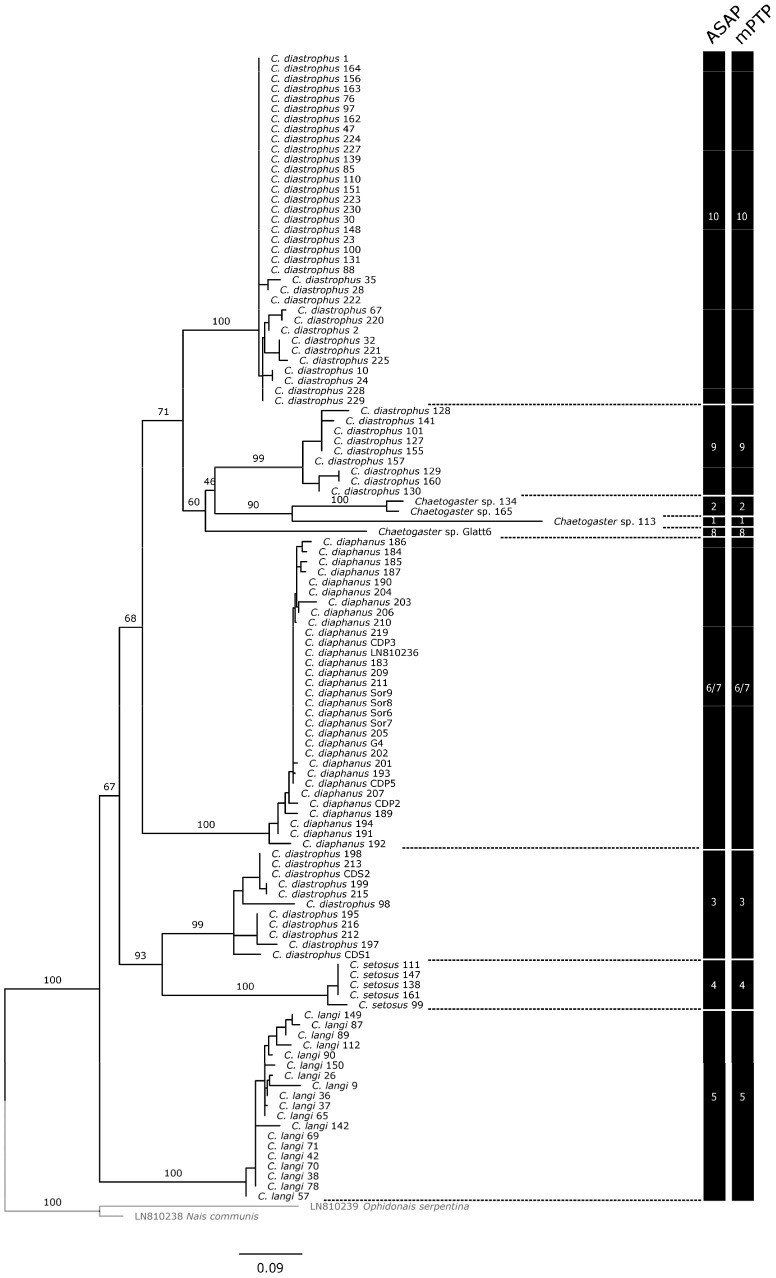
Molecular phylogeny constructed using the maximum likelihood method and ITS2 gene fragment of *Chaetogaster* specimens. Partitions at the right side of the figure represent the results of the species delimitation analyses with single-locus methods (ASAP, mPTP). For comparison purposes, the MOTU numbers correspond to those used for the MOTUs identified from the COI. Numbers at nodes are ultrafast bootstrap values (BV). Nodes were considered supported if BVs were higher or equal to 90 [19]. For the sake of clarity, BVs are not shown within delimited MOTUs.

Delineations based on ITS2 and 28S confirm these results, except for the morphospecies *C. diaphanus*, which appears as a single lineage, so that only 9 MOTUs are delineated based on nuclear genes (Figure 2 and Figure S1).

ITS2 produces identical delineations with both ASAP and mPTP. These delimitations also correspond to strongly supported singletons or clades in the ML tree (BV: 99–100). A barcoding gap remains, albeit narrower than that observed on the basis of COI (p-distances, JC69: 6–8%; K2P: 8–10%).

28S produced less consistent results between methods than ITS2. A discordance is observed in the ASAP analyses depending on whether the evolutionary model is p-distances and K2P (9 MOTUs identified) or JC69 (8 MOTUs by merging MOTU7 and MOTU8). However, it should be noted that, in the latter case, the second-best solution from the ASAP analysis delimits 9 MOTUs again. These MOTUs correspond to singletons or clades strongly supported in the ML tree (BV: 98–100), with the exception of M10, which forms a clade supported by a BV of 94. In contrast, the mPTP approach leads to inconsistent results, both in relation to the ASAP analyses and in relation to the clades identified by the ML analysis: the specimens identified as *C. setosus* (MOTU4) are split between two lineages despite their genetic proximity, and in addition to the lumping of MOTU6 and MOTU7 already observed with the ITS2, a new grouping occurs between MOTU5, MOTU8, MOTU9, and MOTU10, which appear as a single lineage; *C. langi* no longer appears as a distinct species. There is no visible barcoding gap.

### 3.3. Distance Analyses

In COI, the maximum intra-MOTU variations were low (max. 3.9%) and the minimal inter-MOTU variations were high (11.5–14.9%) except for the two MOTUs of *C. diaphanus* (9.6%) (Table 1). In ITS2, the maximum intra-MOTU variations were between 2.7% and 6.2% and the minimal inter-MOTU variations were between 7.5% and 13.9%.

### 3.4. Morphological vs. Molecular Identifications

**Figure 3:**
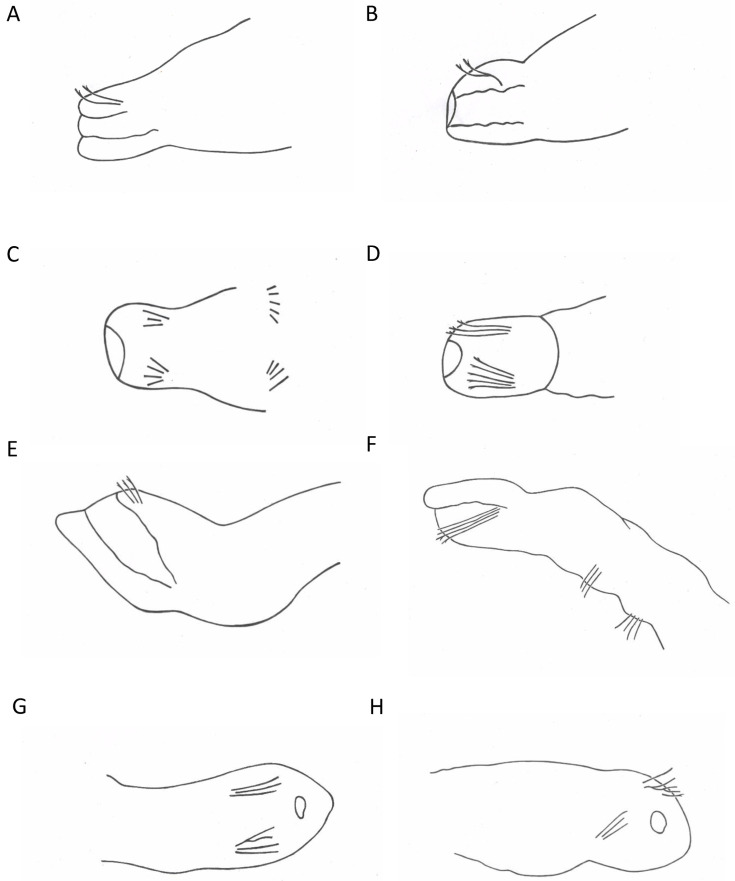
Drawings of anterior parts of *C. langi* (**A**–**D**) and *C. diastrophus* (**E**–**H**) showing an absence or a vestigial state of the prostomium in *C. langi* and a well-developed prostomium in *C. diastrophus* ((**A**) = isolate No 150, (**B**) = isolate No 13, (**C**) = isolate No 87, (**D**) = isolate No 25, (**E**) = isolate No 85, (**F**) = isolate No 156, (**G**) = isolate No 164, and (**H**) = isolate No 23); lateral view in (**A**,**B**,**E**,**F**), ventral view in (**C**,**D**,**G**,**H**); the black arrows show the buccal cavity of the specimens; the blue arrows show the prostomium of the specimens or where it would be if it was present.

As we morphologically identified some specimens as *C. langi* while they belonged to a *C. diastrophus* lineage and inversely, we concluded that the morphological features attributed to *C. langi* were, in practice, not entirely reliable and that identification errors were possible. Indeed, fixation can cause a deformation or flattening of the prostomium, and the position on the slide of a *C. langi* specimen can falsely suggest that the specimen has a developed prostomium. We, therefore, suggest that doubtful specimens of *C. langi*/*C. diastrophus* should be identified as *Chaetogaster* sp. or that genetic analysis carried out when possible. However, the morphological features specific to *C. diastrophus* and *C. langi* (Figure 3) can be verified on most specimens of each morphological group, so we consider that *C. langi* is significantly different from *C. diastrophus* and that a specific lineage can be attributed to this species.

*Chaetogaster* sp. MOTU1 and MOTU2 were characterized morphologically based on observations of only three specimens. They had a size close to the one of *C. langi*, so smaller than the one of *C. diastrophus*. However, we could not identify them as *C. langi* as they seemed to possess a developed prostomium. For *Chaetogaster* MOTU8, no voucher was available (entire specimen sequenced), so it could not be attributed to morphospecies. We sequenced subsequently 11 additional specimens (No 220–230) from site Dws4 in the Glatt River to obtain a voucher of this lineage but none of them belonged to it. In addition, we could not distinguish morphologically the lineages within the morphogroup *C. diastrophus*, which seemed completely cryptic.

### 3.5. Taxonomy

Here, we describe the three *C. diastrophus* MOTUs 3, 9, and 10 and the *Chaetogaster* spp. MOTUs 1–2 as new species (cf. Discussion section) (Table 2). We also attribute a lineage to *C. langi* (MOTU 5) and *C. setosus* (MOTU 4) and describe material corresponding to *C. diaphanus* (MOTUs 6–7) and to the new lineage of *Chaetogaster* sp. MOTU8, for which no anterior part was preserved.

**Morphological group *Chaetogaster diastrophus* (MOTUs 3, 9 and 10)**

Length 0.6–1.2 mm, diameter 0.11–0.16 mm (head). Prostomium present. Fusion of the first five segments forming a head containing a muscular pharynx, buccal cavity not large to large. Ventral chaetae bifid, absent in segments III–V; dorsal chaetae completely lacking.

***Chaetogaster communis*** **sp. nov. Vivien, Lafont & Martin (MOTU10)**

*Holotype*: Isolate No 1; anterior part of the body mounted on a microscopic slide in a medium containing glycerol, lactic acid, and Mowiol, specimen voucher: GBIFCH 1222926; DNA voucher stored in buffer at −20 °C at the Ecotox Center in Lausanne.

*Type locality*: Flawil (St. Gallen, Switzerland), 47.419157° N 9.195899° E.

*Paratypes*: Isolates No 2, 10, 21, 23, 24, 28, 30, 32, 35, 47, 67, 76, 85, 88, 97, 100, 110, 131, 139, 148, 151, 156, 162, 163, 164, 220–230 (geographical coordinates in Table 2 and voucher references in Table S1).

*Etymology*: Named “communis” (Latin for “common”) as it seems frequent according to the data from the present study.

*Distribution (previous studies, GenBank data)*: Reported in Switzerland (Geneva area), Sweden, and Norway.

*Diagnostic molecular characters:* The species can be distinguished from other *Chaetogaster* species on the basis of COI, ITS2 and 28S DNA sequences (Table S1). GenBank accession numbers for COI: PP996388-PP996389, PP996393, PP996396-PP996398, PP996402, PP996404, PP996406, PP996408, PP996415, PP996421, PP996423, PP996426, PP996429, PP996432, PP996434, PP996442, PP996446, PP996451, PP996454, PP996456, PP996461, PP996511-PP996521, PQ198874; ITS2: PQ013378-PQ013379, PQ013381-PQ013383, PQ013385-PQ013388, PQ013393, PQ013396, PQ013400, PQ013402, PQ013404, PQ013407, PQ013410, PQ013412, PQ013420, PQ013423, PQ013427, PQ013430, PQ013432, PQ013436-PQ013438, PQ013481-PQ013490; 28S: PQ013492, PQ013497, PQ013499, PQ013506, PQ013510, PQ013515, PQ013518, PQ013521-PQ013523.

***Chaetogaster fluvii*** **sp. nov. Vivien, Lafont & Martin (MOTU3)**

*Holotype*: Isolate CDS1; anterior part of the body mounted on a microscopic slide in a medium containing glycerol, lactic acid, and Mowiol, specimen voucher: GBIFCH 1223004; DNA voucher stored in buffer at −20 °C at the Ecotox Center in Lausanne.

*Type locality*: Muri (Aargau, Switzerland), 47.305217° N 8.327193° E

*Paratypes: Isolates:* CDS2, No 98, 195, 196, 197, 198, 199, 200, 212, 213, 214, 215, 216 (geographical coordinates in Table 2 and voucher references in Table S1).

*Etymology*: Named “fluvii” (Latin for “of river”) as this species was found in streams

Distribution (previous studies, GenBank data): Reported in Sweden, USA, and Norway.

*Diagnostic molecular characters*: The species can be distinguished from other *Chaetogaster* species on the basis of COI, ITS2, and 28S DNA sequences (Table S1). GenBank accession numbers for COI: PP996430, PP996465-PP996466, PP996489-PP996494, PP996505-PP996509; ITS2: PQ013408, PQ013440-PQ013441, PQ013462-PQ013465, PQ013476-PQ013479; 28S: PQ013495, PQ013525-PQ013526, PQ013544-PQ013545, PQ013555-PQ013556.

***Chaetogaster fluminis*** **sp. nov. Vivien, Lafont & Martin (MOTU9)**

*Holotype*: Isolate No 5; anterior part of the body mounted on a microscopic slide in a medium containing glycerol, lactic acid, and Mowiol, specimen voucher: GBIFCH 1222928; DNA voucher stored in buffer at −20 °C at the Ecotox Center in Lausanne.

*Type locality*: Flawil (St. Gallen, Switzerland), 47.419157° N 9.195899° E.

*Paratypes*: Isolates No 34, 86, 101, 127, 128, 129, 130, 141, 155, 157, 159, 160 (geographical coordinates in Table 2 and voucher references in Table S1).

*Etymology*: Named “fluminis” (Latin for “of river”) as this species was found in streams.

*Distribution (previous studies, GenBank data)*: Reported in USA, Canada, Sweden, Norway, United Kingdom, and Italy. 

*Diagnostic molecular characters*: The species can be distinguished from other *Chaetogaster* species on the basis of COI, ITS2, and 28S DNA sequences (Table S1). GenBank accession numbers for COI: PP996390, PP996407, PP996424, PP996433, PP996438-PP996441, PP996448, PP996455, PP996457, PP996459; ITS2: PQ013411, PQ013416-PQ013419, PQ013424, PQ013431, PQ013433-PQ013434; 28S: PQ013498, PQ013503-PQ013505, PQ013512, PQ013519.

**Morphological group *Chaetogaster* spp. (MOTUs 1–2)**

Length 0.5–0.6 mm, diameter 0.07–0.1 mm (head). Prostomium present. Fusion of the first five segments forming a head containing a muscular pharynx, buccal cavity not large to large. Ventral chaetae bifid, absent in segments III-V; dorsal chaetae completely lacking.

***Chaetogaster suzensis*** **sp. nov. Vivien, Lafont & Martin (MOTU1)**

*Holotype*: Isolate No 113; anterior part of the body mounted on a microscopic slide in a medium containing glycerol, lactic acid, and Mowiol, specimen voucher: GBIFCH 1222976; DNA voucher stored in buffer at −20 °C at the Ecotox Center in Lausanne.

*Type locality*: Villeret (Bern, Switzerland), 47.152502° N 7.014924° E.

*Paratypes*: None.

*Etymology*: Named after the stream (Suze River) in which the holotype was found.

*Distribution (previous studies)*: -

*Diagnostic molecular characters*: The species can be distinguished from other *Chaetogaster* species on the basis of COI, ITS2, and 28S DNA sequences (Table S1). GenBank accession number for COI: PP996437; ITS2:PQ013415; 28S: PQ013502.

***Chaetogaster sorgensis*** **sp. nov. Vivien, Lafont & Martin (MOTU2)**

*Holotype*: Isolate No 134; anterior part of the body mounted on a microscopic slide in a medium containing glycerol, lactic acid, and Mowiol, specimen voucher: GBIFCH 1222983; DNA voucher stored in buffer at −20 °C at the Ecotox Center in Lausanne.

*Type locality*: Ecublens (Vaud, Switzerland), geographical coordinates: 46.522661° N 6.573581° E.

*Paratypes*: Isolate No 165 (geographical coordinates in Table 2 and voucher reference in Table S1).

*Etymology*: Named after the stream (Sorge River) in which the holotype was found.

*Distribution (previous studies, GenBank data)*: Reported in Sweden and Norway.

*Diagnostic molecular characters*: The species can be distinguished from other *Chaetogaster* species on the basis of COI, ITS2, and 28S DNA sequences (Table S1). GenBank accession numbers for COI: PP996444, PP996464; ITS2: PQ013421, PQ013439; 28S: PQ013508, PQ013524.

***Chaetogaster langi* (MOTU5)**

*Material examined*: Isolate No 7, 9, 12, 13, 25, 26, 27, 29, 31, 36–40, 42, 57, 65, 69–71, 78, 87, 89, 90, 112, 132, 140, 142, 149, 150 (geographical coordinates in Table 2 and voucher references in Table S1).

*Diagnostic molecular characters*: The species can be distinguished from other *Chaetogaster* species on the basis of COI, ITS2, and 28S DNA sequences (Table S1). GenBank accession numbers for COI: PP996391-PP996392, PP996394-PP996395, PP996399-PP996401, PP996403, PP996405, PP996409-PP996414, PP996416-PP996420, PP996422, PP996425, PP996427-PP996428, PP996436, PP996443, PP996447, PP996449, PP996452-PP996453; ITS2: PQ013380, PQ013384, PQ013389-PQ013392, PQ013394-PQ013395, PQ013397-PQ013399, PQ013401, PQ013403, PQ013405-PQ013406, PQ013414, PQ013425, PQ013428, PQ013429; 28S: PQ013491, PQ013493-PQ013494, PQ013501, PQ013507, PQ013511, PQ013513, PQ013516-PQ013517.

***Chaetogaster setosus* (MOTU4)**

*Material examined*: Isolates No 99, 111, 138, 147, 161 (geographical coordinates in Table 2 and voucher references in Table S1).

*Diagnostic molecular characters*: The species can be distinguished from other *Chaetogaster* species on the basis of COI, ITS2, and 28S DNA sequences (Table S1). GenBank accession numbers for COI: PP996431, PP996435, PP996445, PP996450, PP996460; ITS2: PQ013409, PQ013413, PQ013422, PQ013426, PQ013435; 28S: PQ013496, PQ013500, PQ013509, PQ013514, PQ013520.

***Chaetogaster diaphanus* (MOTUs 6–7)**

*Material examined:* Isolates Sor6-9; G4; CDP1-5, No 183–187, 189–194, 201–207, 209–211, 219 (geographical coordinates in Table 2 and voucher references in Table S1).

*Diagnostic molecular characters*: The species can be distinguished from other *Chaetogaster* species on the basis of COI, ITS2, and 28S DNA sequences (Table S1). GenBank accession numbers for COI: PP996467-PP996471, PP996473-PP996476, PP996478-PP996488, PP996495-PP996504, PP996510; ITS2: PQ013442-PQ013449, PQ013451-PQ013461, PQ013466-PQ013475; 28S: PQ013527-PQ013532, PQ013534-PQ013543, PQ013546- PQ013554, PQ013557.

***Chaetogaster* sp. with no morphological description (MOTU8)**

*Material examined*: Isolate Glatt6 (geographical coordinates in Table 2).

*Diagnostic molecular characters*: The species can be distinguished from other *Chaetogaster* species on the basis of COI, ITS2, and 28S DNA sequences (Table S1). GenBank accession number for COI: PP996477; ITS2: PQ013450; 28S: PQ013533.

## 4. Discussion

### 4.1. Species Delimitations and Chaetogaster Diversity in Switzerland 

It is generally accepted that as a rule of thumb in clitellates, if two clusters delimited on the basis of the COI gene differ from each other by more than 10% of uncorrected genetic distances, they are likely to belong to different species, and if they differ by less than 5%, they are likely to belong to one species [30] (but see Liu et al. [31] for exceptions within the *Limnodrilus hoffmeisteri* species complex). The different approaches and genes used yielded largely congruent results and confirmed the delineation obtained by ASAP with the COI gene, except for MOTUs 6 and 7, grouped together in all analyses based on nuclear genes and the mPTP analysis of 28S sequences.

Incomplete lineage sorting (ILS) and/or introgression are the most common factors invoked to explain phylogenetic discordance between nuclear and mitochondrial markers [32,33]. Distinguishing between the two processes can be a challenging task [34] and is out of the scope of the present study. Although ILS can affect both nuclear and mitochondrial markers, the lack of recombination in mtDNA means that the genetic variation in mtDNA tends to coalesce more rapidly than in nuclear DNA, suggesting that MOTU6 and MOTU7 are possible recent separately evolving lineages—in other words, potential distinct species according to the de Queiroz’s species concept [35].

By delimiting only 6 MOTUs, by merging MOTUs identified in the other analyses (MOTUs 6, 7; 5, 8, 9, 10), and despite the splitting of MOTU4 into two distinct lineages, the mPTP analysis of the 28S sequences clearly stands out from the other results. The splitting of MOTU4 into two lineages is clearly an artifact, given the genetic proximity between the 28S sequences that are split. Usually, mPTP is considered superior to its previous variants (PTP, bPTP) because it produces more taxonomically congruent delineations [30]. However, it is possible that the lower variability of 28S compared with COI and ITS2 partially explains this result. Indeed, Dellicour and Flot [25] showed that PTP and bPTP tend to produce more overlumping when the mutation rate of the markers is low. 

*Chaetogaster* sp. MOTUs 1–2, considered morphologically as possibly intermediate between *C. diastrophus* and *C. langi*, were, on the phylogenetic analysis of COI and 28S, early-branching, which shows an important genetic divergence between these two lineages and the rest of the *Chaetogaster* lineages. *Chaetogaster sp.* MOTU2, corresponding to sp. 1 in Mack et al. [2], was also early-branching on a concatenated tree based on COI, ITS2, and H3 markers built by these authors. The COI, ITS2, and 28S data suggest that *Chaetogaster* sp. MOTU8 is a cryptic species of *C. diastrophus.* But we cannot exclude that it belongs to the morphospecies *C. limnaei* or *C. parvus* (anterior part not preserved).

As ITS2 and 28S data suggested that all *C. diaphanus* MOTUs 6–7 sequences formed only one lineage, we decided to regroup all sequences of this morphogroup in a unique species, as a conservative measure. The genetic divergence in COI barely reached the threshold of 10% used to distinguish clitellate species, so the distinction based on COI data only was already doubtful. Mack et al. [2] considered these two MOTUs as significantly different based on the analysis of COI, the mitochondrial marker 16S rDNA, and the nuclear region H3. The ITS2 sequences obtained by Mack et al. [2] also showed a distinction between these MOTUs, which is in contradiction with our ITS2 data. This discordance is difficult to understand, and we cannot exclude that the ITS2 sequences provided by Mack et al. [2] for MOTU sp. 3 (=our MOTU7) correspond, in fact, to specimens other than those of this MOTU.

*Chaetogaster langi* was considered by some authors as a synonym of *C. diastrophus* [2]. We were able to attribute a DNA barcode to the morphospecies *C. langi* and demonstrate that it was morphologically significantly different from *C. diastrophus.* Mack et al. [2] found this lineage but did not describe it as belonging to *C. langi*, probably because the small size of the *Chaetogaster* specimens required the extraction of DNA from whole specimens, making it impossible to go back to the morphological study of the lineages.

In addition, Mack et al. [2] did not mention that one of the *C. diastrophus* lineages they found could belong to *Chaetogaster parvus* Poitner, 1914, probably because this species is considered by some authors as invalid or a synonym of *C. langi* [10,36] despite the mention of its presence in France [37] and Switzerland and the fact that it can be easily differentiated from *C. langi* or *C. diastrophus* by the form of the crotchets [37,38]. Finally, Mack et al. [2] discussed the possibility that one of the two MOTUs of *C. diaphanus* could be confined to North America (sp. 3) and the other to Europe (sp. 4). But in the present study, the MOTU (MOTU7)—supposed to be present only in North America—was abundant in one stream, which suggests that it is also well represented in Europe.

In terms of diversity, we observed a large number of *Chaetogaster* lineages in/at a low number of streams and sites. These results suggest that we only collected the part of the *Chaetogaster* lineages present in Switzerland and that a larger number of *Chaetogaster* species could be found in this small territory. The phylogenetic and morphological analyses suggest that the lineages *Chaetogaster* sp. MOTUs 1–2 could correspond to a new morphological group (morphospecies). However, this observation should be confirmed based on the morphological analysis of more specimens belonging to these lineages.

### 4.2. Description of New Chaetogaster Species

It is widely acknowledged that cryptic species within morphospecies are frequent in metazoans and that they should be considered distinct species [39]. It is now accepted that in many cases, speciation in metazoans can occur without any (visible) morphological modifications [40]. However, there is still no consensus on the criteria required to assign the status of nominal species to morphologically indistinguishable lineages. Many taxonomists agree that taxonomic descriptions should be integrative, i.e., consider all available data in the literature (morphology, molecular sequences, behavior, ecology, reproductive isolation, etc.) to establish if the different lineages within the same morphospecies are distinct species [40,41]. The taxonomists should, however, select some characteristics that could be pertinent to distinguish cryptic species in a particular group [42,43]. For example, behavioral and reproductive isolation experiments could be conducted on terrestrial oligochaetes, such as lumbricids [44,45,46]. However, these experiments are not feasible for rare aquatic oligochaete species, which are difficult or nearly impossible to isolate and cultivate. Such experiments, for example, could not be performed on cryptic species of *Chaetogaster*. Indeed, to attribute a lineage to a specimen of this genus, it is necessary to collect at least half of its body for genetic analyses, and *Chaetogaster* specimens do not have the property to regenerate their posterior segments. Given the current state of scientific knowledge and technological capabilities, it is probable that for the most cryptic metazoan species (e.g., worms, insects, etc.) on Earth, only DNA sequences could be used as a tool for distinguishing them. Molecular analyses that have proven to be effective for the identification of species in many invertebrate groups, including annelids (e.g., [47,48,49]) could be sufficient tools to delimit and describe species. 

The fact that cryptic species remain almost always considered MOTUs, OTUs, or putative species and named differently in the different studies leads to a denial of their existence as real species and, consequently, their absence from species inventories and conservation programs [42]. Once their validity is established (based on DNA data and if possible other observations), these cryptic species should consequently be described as nominal species and considered by the scientific community as an integrant part of biodiversity [31,49]. There are no official requirements of the ICZN that the description of species should be based only on morphological grounds [42].

We agree with Zamani et al. [50] and Martinsson and Erséus [49] that the description of species using only DNA data should be avoided and that it should be accompanied by morphological observations. We preserved a voucher for most sequenced specimens and tried to find distinctive characters between the lineages of the same morphospecies and specific features of the lineages identified as *Chaetogaster* spp. 

Currently, the newly described species of *Chaetogaster* could be recognized only using genetic analyses as we could not find any morphological characters, such as the specimen size and the form of prostomium and crotchets specific to each of them. We cannot expect in the short and medium term to characterize each one using the morphology of their reproductive organs as sexually mature *Chaetogaster* specimens are very rarely found. In addition, it is necessary to cut specimens in two to sequence them, so the segments bearing the genital features can be removed or damaged. Therefore, only an examination of live sexually mature specimens and photo acquisitions before sequencing could allow us to differentiate and describe them based on these characters. This procedure would not allow us to preserve a voucher containing the distinctive morphological features, and of course, it is not evident at all that the species of the same morphospecies would show observable differences in the genital apparatus.

The nominal species *C. diastrophus* [51] could potentially correspond to one of the three described lineages of this morphospecies. But its type has probably been lost or cannot be sequenced. In the 19th century, specimens were generally fixed using low-pH formalin and preserved in this medium or in 70% ethanol. In addition, the type could be preserved mounted on a slide in a conservative medium, which could also hamper subsequent genetic analyses. When type material is lost or cannot be used for genetic analyses, assigning a genetic lineage to a nominal species still remains possible as long as new biological material is collected as close as possible to the original type locality [49]. Neotypes can, thus, be designated in compliance with the conditions required by the International Code of Zoological Nomenclature (Art. 75.3), in particular the provision of Article 75.3.6 relating to the original type locality, and genetically characterized. Sampling *C. diastrophus* at the type locality would not definitively determine the lineage of the type specimen as multiple lineages corresponding to this morphospecies may coexist at the same locality. For example, in the present study, the three lineages of *C. diastrophus* were present at site 9 in the Suze River. 

As the *C. diastrophus* specimens of the present study were certainly not sampled at or close to the type locality (which is not mentioned in Gruithuisen [51]), we described each lineage corresponding to this morphospecies as a new species for science. If, in the future, it could be demonstrated that one of these new lineages corresponded to the nominal species, it would be possible to consider the new lineage as a synonym to the nominal species. The nominal species *C. diastrophus* should currently be considered species inquirendae. We recommend that any future studies based solely on a morphological approach should refer to it as species “sensu lato”.

As it has not been demonstrated that the nominal species *C. diaphanus*, *C. setosus*, and *C. langi* contained cryptic species, the lineages obtained in the present study for each of these species can be considered as belonging to the nominal species. If, in the future, cryptic species were detected within these species, we would recommend describing the cryptic lineages as new species for science and considering the nominal species as species inquirendae. The new lineage MOTU8 could not be described as a new species for science as we could not preserve any voucher for this lineage. Even if the phylogenetic analyses suggest that it is an additional cryptic species of *C. diastrophus*, it may belong to the species *C. limnaei* or *C. parvus*.

## 5. Conclusions

Here, we reported nine *Chaetogaster* lineages based on specimens collected at a few sites in a small territory and we were able to genetically characterize the species of *Chaetogaster langi* and *Chaetogaster setosus*. We described five new *Chaetogaster* species, among them three within the nominal species *Chaetogaster diastrophus* and two *Chaetogaster* spp., which could possibly belong to a new morphological group. A large number of cryptic species of aquatic oligochaetes have been reported so far but are still not considered an integrant part of biodiversity as none was described as a new species for science. Several cryptic species (lineages) were reported, for example, within *Limnodrilus hoffmeisteri* [31], *Globulidrilus riparius* [52], *Nais communis* [53], and *Haplotaxis gordioides* [54]. We believe that these new *Chaetogaster* species validated based on mitochondrial and nuclear data should be fully considered as individual species by the scientific community, especially because it cannot be expected to characterize each of them using morphological features. The genus *Chaetogaster* represents an excellent candidate for initiating descriptions of cryptic species within aquatic oligochaetes. The prospects of the present work are to complete the data of the molecular diversity of this genus in Switzerland and to describe, on a molecular/morphological basis, the future new *Chaetogaster* cryptic species that we will probably find. Special effort will be made to barcode *C. parvus* to confirm its existence as a separate species. 

## Figures and Tables

**Table 1:** Percentages of maximal intra-MOTU and minimal inter-MOTU variations (p-distances) in COI and ITS2 of each MOTU of *Chaetogaster* found in the present study, with an indication of the correspondence of each MOTU to the species (=lineages) described by Mack et al. [2].

	No of Species Found by [2]	Maximal Intra-MOTU Variation (%) in COI	Minimal Inter-MOTU Variation (%) in COI	Maximal Intra-MOTU Variation (%) in ITS2	Minimal Inter-MOTU Variation (%) in ITS2
*C. diaphanus* MOTU6	“*C. diaphanus*” sp. 4	2.12	9.57	4.97	7.46
*C. diaphanus* MOTU7	“*C. diaphanus*” sp. 3	0.05	9.57		
*C. diastrophus* MOTU10	“*C. diastrophus*” sp. 11	3.87	11.45	4.48	9.60
*C. diastrophus* MOTU3	“*C. diastrophus*” sp. 8	2.74	14.35	6.21	7.46
*C. diastrophus* MOTU9	“*C. diastrophus*” sp. 12	2.89	11.45	5.58	11.59
*C. langi* MOTU5	“*C. diastrophus*” sp. 19	0.91	13.67	5.68	10.34
*C. setosus* MOTU4		0.00	12.61	2.73	12.50
*Chaetogaster* sp. MOTU1		NC	14.89	NC	13.86
*Chaetogaster* sp. MOTU2	“*C. diastrophus*” sp. 1	0	12.00	2.78	12.29
*Chaetogaster* sp. MOTU8	“*C. diastrophus*” sp. 18	NC	11.89	NC	9.84

NC = not calculable.

**Table 2:** For each lineage, indication of the new species name (when applicable) and information on geographical coordinates, preservation of material (DNA and voucher); the habitat is the same for all specimens, surface coarse sediments in streams.

Morphospecies/Group	New Species Name	Geographical Coordinates	Material Preservation
*Chaetogaster diaphanus* MOTUs 6–7		Isolates Sor6-9: 46.522661° N, 6.573581° E; Isolate G4: 47.419157° N, 9.195899° E; Isolates CDP1-5, No 201–207, 209–211, 219, No 183–187, 189–194: 47.305217° N, 8.327193° E	DNA voucher of the 32 specimens stored in buffer at −20 °C at the Ecotox Center in Lausanne; anterior part of 30 specimens (all except No 204 and 205) preserved (mounted on slides) in the Muséum cantonal des sciences naturelles of Lausanne.
*Chaetogaster diastrophus* MOTU10	*Chaetogaster communis* sp. nov. Vivien, Lafont & Martin	Isolates No 1, 2, 10, 21: 47.419157° N, 9.195899° E; Isolates No 23, 24, 28, 30, 32, 35, 47, 67, 76, 85: 47.414825° N, 9.198461° E; Isolates No 88, 97, 100, 110, 131, 139, 148, 151, 156, 162, 163, 164: 47.152502° N, 7.014924° E; Isolates No 220–230: 47.43167° N, 9.17485° E	Holotype and paratypes: DNA voucher of the 36 specimens stored in buffer at −20 °C at the Ecotox Center in Lausanne; anterior part of 35 specimens (all except No. 30) preserved (mounted on slides) in the Muséum cantonal des sciences naturelles of Lausanne.
*Chaetogaster diastrophus* MOTU3	*Chaetogaster fluvii* sp. nov. Vivien, Lafont & Martin	Isolates CDS1, CDS2, No 195, 196, 197, 198, 199, 200, 212, 213, 214, 215, 216: 47.305217° N, 8.327193° E; Isolate No 98: 47.152502° N, 7.014924° E	Holotype and paratypes: DNA voucher of the 14 specimens stored in buffer at −20 °C at the Ecotox Center in Lausanne; anterior part of the 14 specimens preserved (mounted on slides) in the Muséum cantonal des sciences naturelles of Lausanne.
*Chaetogaster diastrophus* MOTU9	*Chaetogaster fluminis* sp. nov. Vivien, Lafont & Martin	Isolates No 5: 47.419157° N, 9.195899° E; Isolates No 34, 86: 47.414825° N, 9.198461° E; Isolate No 101: 47.152502° N, 7.014924° E Isolates No 127, 128, 129, 130 46.522661° N, 6.573581° E; Isolates No 141, 155, 157, 159, 160: 47.152502° N, 7.014924° E	Holotype and paratypes: DNA voucher of the 13 specimens stored in buffer at −20 °C at the Ecotox Center in Lausanne; anterior part of 12 specimens (all except No 155) preserved (mounted on slides) in the Muséum cantonal des sciences naturelles of Lausanne.
*Chaetogaster* sp. MOTU1	*Chaetogaster suzensis* sp. nov. Vivien, Lafont & Martin	Isolate No 113: 47.152502° N, 7.014924° E	Holotype: DNA voucher of the specimen stored in buffer at −20 °C at the Ecotox Center in Lausanne; anterior part of the specimen (mounted on a slide) preserved in the Muséum cantonal des sciences naturelles of Lausanne.
*Chaetogaster* sp. MOTU2	*Chaetogaster sorgensis* sp. nov. Vivien, Lafont & Martin	Isolate No 134: 46.522661° N, 6.573581° E; Isolate No 165: 47.152502° N, 7.014924° E	Holotype and paratype: DNA voucher of the 2 specimens stored in buffer at −20 °C at the Ecotox Center in Lausanne; anterior part of the 2 specimens preserved (mounted on slides) in the Muséum cantonal des sciences naturelles of Lausanne.
*Chaetogaster* sp. MOTU8		Isolate Glatt6: 47.43167° N, 9.17485° E	DNA voucher of the specimen (Glatt 6) stored in buffer at −20 °C at the Ecotox Center in Lausanne.
*Chaetogaster langi* MOTU5		Isolates No 7, 9, 12, 13: 47.419157° N, 9.195899 ° E; Isolates No 25, 26, 27, 29, 31, 36–40, 42, 57, 65, 69–71, 78: 47.414825° N, 9.198461° E; Isolates No 87, 89, 90, 112: 47.152502° N, 7.014924° E; Isolate No 132: 46.522661° N, 6.573581° E; Isolates No 140, 142, 149, 150: 47.152502° N, 7.014924° E	DNA voucher of the 30 specimens stored in buffer at −20 °C at the Ecotox Center in Lausanne; anterior part of 27 specimens (all except No 29, 37, and 38) preserved (mounted on slides) in the Muséum cantonal des sciences naturelles of Lausanne.
*Chaetogaster setosus* MOTU4		Isolates No 99, 111, 138, 147, 161: 47.152502° N, 7.014924 ° E	DNA voucher of the 5 specimens stored in buffer at −20 °C at the Ecotox Center in Lausanne; anterior part of the 5 specimens preserved (mounted on slides) in the Muséum cantonal des sciences naturelles of Lausanne.

## Data Availability

The COI, ITS2, and 28S sequences are provided as Supplemental Files S1–S3 and are accessible in GenBank: COI: PP996388-PP996521, PQ198874; ITS2: PQ013378-PQ013490; 28S: PQ013491-PQ013557. The anterior parts of all specimens (vouchers), except isolates Glatt6, Nos 29, 30, 37, 38, 155, 204, 205, are deposited in the “Muséum cantonal des sciences naturelles” of Lausanne (Table S1).

## Supplementary Materials

The following supporting information can be downloaded at: https://www.mdpi.com/article/10.3390/biology13090693/s1, Table S1: Details of each *Chaetogaster* specimen: sampling, morphological identification, GenBank accession numbers (COI, ITS2 and 28S), and specimen voucher reference (Place of deposit: Muséum cantonal des sciences naturelles of Lausanne). Table S2: Mean genetic variations (p-distances) between the MOTUs of the present study (M01–M10) and the lineages found by Mack et al. (2023) (Sp01–Sp24). Figure S1: Molecular phylogeny constructed using the maximum likelihood method and 28S gene fragment of *Chaetogaster* specimens. Partitions at the right side of the figure represent the results of the species delimitation analyses with single-locus methods (ASAP, mPTP). For comparison purposes, the MOTU numbers correspond to those used for the MOTUs identified from the COI. Numbers at nodes are ultrafast bootstrap values (BV). Nodes were considered supported if BVs were higher or equal to 90 [19]. For the sake of clarity, BVs are not shown within delimited MOTUs. File S1: Aligned COI sequences of *Chaetogaster* obtained in the present study. File S2: Aligned ITS2 sequences of *Chaetogaster* obtained in the present study. File S3: Aligned 28S sequences of *Chaetogaster* obtained in the present study.
