# Supplementary material for: The Genus Chaetogaster Baer, 1827 (Annelida, Clitellata) in Switzerland: A First Step toward Cataloguing Its Molecular Diversity and Description of New Species on a DNA Sequence Basis
Source: Biology (Basel). 2024 Sep 4;13(9):693. doi: 10.3390/biology13090693 (PMC11428632; doi:10.3390/biology13090693)
Supplement: Supplementary file 1 [file biology-13-00693-s001.zip › Table_S1.pdf]

Isolate	Morphospecies / Group	Country	Canton	Locality	Site	Stream	Collected by	Collection date	Identified by	GenBank accession number COI	GenBank accession number ITS2	GenBank accession number 28S	Specimen voucher
1	*Chaetogaster diastrophus* MOTU10	CH	St. Gallen	Flawil	Dws2	Glatt River	Benoit Ferrari	November 9, 2022	Régis Vivien	PP996388	PQ013378		GBIFCH 1222926
2	*Chaetogaster diastrophus* MOTU10	CH	St. Gallen	Flawil	Dws2	Glatt River	Benoit Ferrari	November 9, 2022	Régis Vivien	PP996389	PQ013379		GBIFCH 1222927
5	*Chaetogaster diastrophus* MOTU9	CH	St. Gallen	Flawil	Dws2	Glatt River	Benoit Ferrari	November 9, 2022	Régis Vivien	PP996390			GBIFCH 1222928
7	*Chaetogaster langi* MOTU5	CH	St. Gallen	Flawil	Dws2	Glatt River	Benoit Ferrari	November 9, 2022	Régis Vivien	PP996391			GBIFCH 1222929
9	*Chaetogaster langi* MOTU5	CH	St. Gallen	Flawil	Dws2	Glatt River	Benoit Ferrari	November 9, 2022	Régis Vivien	PP996392	PQ013380		GBIFCH 1222930
10	*Chaetogaster diastrophus* MOTU10	CH	St. Gallen	Flawil	Dws2	Glatt River	Benoit Ferrari	November 9, 2022	Régis Vivien	PP996393	PQ013381		GBIFCH 1222931
12	*Chaetogaster langi* MOTU5	CH	St. Gallen	Flawil	Dws2	Glatt River	Benoit Ferrari	November 9, 2022	Régis Vivien	PP996394			GBIFCH 1222932
13	*Chaetogaster langi* MOTU5	CH	St. Gallen	Flawil	Dws2	Glatt River	Benoit Ferrari	November 9, 2022	Régis Vivien	PP996395			GBIFCH 1222933
21	*Chaetogaster diastrophus* MOTU10	CH	St. Gallen	Flawil	Dws2	Glatt River	Benoit Ferrari	November 9, 2022	Régis Vivien	PP996396			GBIFCH 1222934
23	*Chaetogaster diastrophus* MOTU10	CH	St. Gallen	Flawil	Dws1	Glatt River	Benoit Ferrari	November 9, 2022	Régis Vivien	PP996397	PQ013382		GBIFCH 1222935
24	*Chaetogaster diastrophus* MOTU10	CH	St. Gallen	Flawil	Dws1	Glatt River	Benoit Ferrari	November 9, 2022	Régis Vivien	PP996398	PQ013383		GBIFCH 1222936
25	*Chaetogaster langi* MOTU5	CH	St. Gallen	Flawil	Dws1	Glatt River	Benoit Ferrari	November 9, 2022	Régis Vivien	PP996399			GBIFCH 1222937
26	*Chaetogaster langi* MOTU5	CH	St. Gallen	Flawil	Dws1	Glatt River	Benoit Ferrari	November 9, 2022	Régis Vivien	PP996400	PQ013384		GBIFCH 1222938
27	*Chaetogaster langi* MOTU5	CH	St. Gallen	Flawil	Dws1	Glatt River	Benoit Ferrari	November 9, 2022	Régis Vivien	PP996401			GBIFCH 1222939
28	*Chaetogaster diastrophus* MOTU10	CH	St. Gallen	Flawil	Dws1	Glatt River	Benoit Ferrari	November 9, 2022	Régis Vivien	PP996402	PQ013385		GBIFCH 1222940
29	*Chaetogaster langi* MOTU5	CH	St. Gallen	Flawil	Dws1	Glatt River	Benoit Ferrari	November 9, 2022	Régis Vivien	PP996403			
30	*Chaetogaster diastrophus* MOTU10	CH	St. Gallen	Flawil	Dws1	Glatt River	Benoit Ferrari	November 9, 2022	Régis Vivien	PP996404	PQ013386		
31	*Chaetogaster langi* MOTU5	CH	St. Gallen	Flawil	Dws1	Glatt River	Benoit Ferrari	November 9, 2022	Régis Vivien	PP996405			GBIFCH 1222943
32	*Chaetogaster diastrophus* MOTU10	CH	St. Gallen	Flawil	Dws1	Glatt River	Benoit Ferrari	November 9, 2022	Régis Vivien	PP996406	PQ013387		GBIFCH 1222944
34	*Chaetogaster diastrophus* MOTU9	CH	St. Gallen	Flawil	Dws1	Glatt River	Benoit Ferrari	November 9, 2022	Régis Vivien	PP996407			GBIFCH 1222945
35	*Chaetogaster diastrophus* MOTU10	CH	St. Gallen	Flawil	Dws1	Glatt River	Benoit Ferrari	November 9, 2022	Régis Vivien	PP996408	PQ013388		GBIFCH 1222946
36	*Chaetogaster langi* MOTU5	CH	St. Gallen	Flawil	Dws1	Glatt River	Benoit Ferrari	November 9, 2022	Régis Vivien	PP996409	PQ013389		GBIFCH 1222947

Isolate	Morphospecies / Group	Country	Canton	Locality	Site	Stream	Collected by	Collection date	Identified by	GenBank accession number COI	GenBank accession number ITS2	GenBank accession number 28S	Specimen voucher
37	*Chaetogaster langi* MOTU5	CH	St. Gallen	Flawil	Dws1	Glatt River	Benoit Ferrari	November 9, 2022	Régis Vivien	PP996410	PQ013390		
38	*Chaetogaster langi* MOTU5	CH	St. Gallen	Flawil	Dws1	Glatt River	Benoit Ferrari	November 9, 2022	Régis Vivien	PP996411	PQ013391		
39	*Chaetogaster langi* MOTU5	CH	St. Gallen	Flawil	Dws1	Glatt River	Benoit Ferrari	November 9, 2022	Régis Vivien	PP996412			GBIFCH 1222950
40	*Chaetogaster langi* MOTU5	CH	St. Gallen	Flawil	Dws1	Glatt River	Benoit Ferrari	November 9, 2022	Régis Vivien	PP996413			GBIFCH 1222951
42	*Chaetogaster langi* MOTU5	CH	St. Gallen	Flawil	Dws1	Glatt River	Benoit Ferrari	November 9, 2022	Régis Vivien	PP996414	PQ013392		GBIFCH 1222952
47	*Chaetogaster diastrophus* MOTU10	CH	St. Gallen	Flawil	Dws1	Glatt River	Benoit Ferrari	November 9, 2022	Régis Vivien	PP996415	PQ013393		GBIFCH 1222953
57	*Chaetogaster langi* MOTU5	CH	St. Gallen	Flawil	Dws2	Glatt River	Benoit Ferrari	November 9, 2022	Régis Vivien	PP996416	PQ013394		GBIFCH 1222954
65	*Chaetogaster langi* MOTU5	CH	St. Gallen	Flawil	Dws1	Glatt River	Benoit Ferrari	November 9, 2022	Régis Vivien	PP996417	PQ013395		GBIFCH 1222955
67	*Chaetogaster diastrophus* MOTU10	CH	St. Gallen	Flawil	Dws1	Glatt River	Benoit Ferrari	November 9, 2022	Régis Vivien	PQ198874	PQ013396		GBIFCH 1222956
69	*Chaetogaster langi* MOTU5	CH	St. Gallen	Flawil	Dws1	Glatt River	Benoit Ferrari	November 9, 2022	Régis Vivien	PP996418	PQ013397		GBIFCH 1222957
70	*Chaetogaster langi* MOTU5	CH	St. Gallen	Flawil	Dws1	Glatt River	Benoit Ferrari	November 9, 2022	Régis Vivien	PP996419	PQ013398		GBIFCH 1222958
71	*Chaetogaster langi* MOTU5	CH	St. Gallen	Flawil	Dws1	Glatt River	Benoit Ferrari	November 9, 2022	Régis Vivien	PP996420	PQ013399		GBIFCH 1222959
76	*Chaetogaster diastrophus* MOTU10	CH	St. Gallen	Flawil	Dws1	Glatt River	Benoit Ferrari	November 9, 2022	Régis Vivien	PP996421	PQ013400		GBIFCH 1222960
78	*Chaetogaster langi* MOTU5	CH	St. Gallen	Flawil	Dws1	Glatt River	Benoit Ferrari	November 9, 2022	Régis Vivien	PP996422	PQ013401		GBIFCH 1222961
85	*Chaetogaster diastrophus* MOTU10	CH	St. Gallen	Flawil	Dws1	Glatt River	Benoit Ferrari	November 9, 2022	Régis Vivien	PP996423	PQ013402		GBIFCH 1222962
86	*Chaetogaster diastrophus* MOTU9	CH	St. Gallen	Flawil	Dws1	Glatt River	Benoit Ferrari	November 9, 2022	Régis Vivien	PP996424			GBIFCH 1222963
87	*Chaetogaster langi* MOTU5	CH	Bern	Villeret	9	Suze River	Pascal Mulattieri	April 7, 2023	Régis Vivien	PP996425	PQ013403	PQ013491	GBIFCH 1222964
88	*Chaetogaster diastrophus* MOTU10	CH	Bern	Villeret	9	Suze River	Pascal Mulattieri	April 7, 2023	Régis Vivien	PP996426	PQ013404	PQ013492	GBIFCH 1222965
89	*Chaetogaster langi* MOTU5	CH	Bern	Villeret	9	Suze River	Pascal Mulattieri	April 7, 2023	Régis Vivien	PP996427	PQ013405	PQ013493	GBIFCH 1222966
90	*Chaetogaster langi* MOTU5	CH	Bern	Villeret	9	Suze River	Pascal Mulattieri	April 7, 2023	Régis Vivien	PP996428	PQ013406	PQ013494	GBIFCH 1222967
97	*Chaetogaster diastrophus* MOTU10	CH	Bern	Villeret	9	Suze River	Pascal Mulattieri	April 7, 2023	Régis Vivien	PP996429	PQ013407		GBIFCH 1222968
98	*Chaetogaster diastrophus* MOTU3	CH	Bern	Villeret	9	Suze River	Pascal Mulattieri	April 7, 2023	Régis Vivien	PP996430	PQ013408	PQ013495	GBIFCH 1222969

Isolate	Morphospecies / Group	Country	Canton	Locality	Site	Stream	Collected by	Collection date	Identified by	GenBank accession number COI	GenBank accession number ITS2	GenBank accession number 28S	Specimen voucher
99	*Chaetogaster setosus* MOTU4	CH	Bern	Villeret	9	Suze River	Pascal Mulattieri	April 7, 2023	Régis Vivien	PP996431	PQ013409	PQ013496	GBIFCH 1222970
100	*Chaetogaster diastrophus* MOTU10	CH	Bern	Villeret	9	Suze River	Pascal Mulattieri	April 7, 2023	Régis Vivien	PP996432	PQ013410	PQ013497	GBIFCH 1222971
101	*Chaetogaster diastrophus* MOTU9	CH	Bern	Villeret	9	Suze River	Pascal Mulattieri	April 7, 2023	Régis Vivien	PP996433	PQ013411	PQ013498	GBIFCH 1222972
110	*Chaetogaster diastrophus* MOTU10	CH	Bern	Villeret	9	Suze River	Pascal Mulattieri	April 7, 2023	Régis Vivien	PP996434	PQ013412	PQ013499	GBIFCH 1222973
111	*Chaetogaster setosus* MOTU4	CH	Bern	Villeret	9	Suze River	Pascal Mulattieri	April 7, 2023	Régis Vivien	PP996435	PQ013413	PQ013500	GBIFCH 1222974
112	*Chaetogaster langi* MOTU5	CH	Bern	Villeret	9	Suze River	Pascal Mulattieri	April 7, 2023	Régis Vivien	PP996436	PQ013414	PQ013501	GBIFCH 1222975
113	*Chaetogaster* sp. MOTU1	CH	Bern	Villeret	9	Suze River	Pascal Mulattieri	April 7, 2023	Régis Vivien	PP996437	PQ013415	PQ013502	GBIFCH 1222976
127	*Chaetogaster diastrophus* MOTU9	CH	Vaud	Ecublens	UNIL-Sorge	Sorge River	Régis Vivien	April 2020	Régis Vivien	PP996438	PQ013416		GBIFCH 1222977
128	*Chaetogaster diastrophus* MOTU9	CH	Vaud	Ecublens	UNIL-Sorge	Sorge River	Régis Vivien	April 2020	Régis Vivien	PP996439	PQ013417	PQ013503	GBIFCH 1222978
129	*Chaetogaster diastrophus* MOTU9	CH	Vaud	Ecublens	UNIL-Sorge	Sorge River	Régis Vivien	April 2020	Régis Vivien	PP996440	PQ013418	PQ013504	GBIFCH 1222979
130	*Chaetogaster diastrophus* MOTU9	CH	Vaud	Ecublens	UNIL-Sorge	Sorge River	Régis Vivien	April 2020	Régis Vivien	PP996441	PQ013419	PQ013505	GBIFCH 1222980
131	*Chaetogaster diastrophus* MOTU10	CH	Vaud	Ecublens	UNIL-Sorge	Sorge River	Régis Vivien	April 2020	Régis Vivien	PP996442	PQ013420	PQ013506	GBIFCH 1222981
132	*Chaetogaster langi* MOTU5	CH	Vaud	Ecublens	UNIL-Sorge	Sorge River	Régis Vivien	April 2020	Régis Vivien	PP996443		PQ013507	GBIFCH 1222982
134	*Chaetogaster* sp. MOTU2	CH	Vaud	Ecublens	UNIL-Sorge	Sorge River	Régis Vivien	April 2020	Régis Vivien	PP996444	PQ013421	PQ013508	GBIFCH 1222983
138	*Chaetogaster setosus* MOTU4	CH	Bern	Villeret	9	Suze River	Pascal Mulattieri	April 7, 2023	Régis Vivien	PP996445	PQ013422	PQ013509	GBIFCH 1222984
139	*Chaetogaster diastrophus* MOTU10	CH	Bern	Villeret	9	Suze River	Pascal Mulattieri	April 7, 2023	Régis Vivien	PP996446	PQ013423	PQ013510	GBIFCH 1222985
140	*Chaetogaster langi* MOTU5	CH	Bern	Villeret	9	Suze River	Pascal Mulattieri	April 7, 2023	Régis Vivien	PP996447		PQ013511	GBIFCH 1222986
141	*Chaetogaster diastrophus* MOTU9	CH	Bern	Villeret	9	Suze River	Pascal Mulattieri	April 7, 2023	Régis Vivien	PP996448	PQ013424	PQ013512	GBIFCH 1222987
142	*Chaetogaster langi* MOTU5	CH	Bern	Villeret	9	Suze River	Pascal Mulattieri	April 7, 2023	Régis Vivien	PP996449	PQ013425	PQ013513	GBIFCH 1222988
147	*Chaetogaster setosus* MOTU4	CH	Bern	Villeret	9	Suze River	Pascal Mulattieri	April 7, 2023	Régis Vivien	PP996450	PQ013426	PQ013514	GBIFCH 1222989
148	*Chaetogaster diastrophus* MOTU10	CH	Bern	Villeret	9	Suze River	Pascal Mulattieri	April 7, 2023	Régis Vivien	PP996451	PQ013427	PQ013515	GBIFCH 1222990
149	*Chaetogaster langi* MOTU5	CH	Bern	Villeret	9	Suze River	Pascal Mulattieri	April 7, 2023	Régis Vivien	PP996452	PQ013428	PQ013516	GBIFCH 1222991

Isolate	Morphospecies / Group	Country	Canton	Locality	Site	Stream	Collected by	Collection date	Identified by	GenBank accession number COI	GenBank accession number ITS2	GenBank accession number 28S	Specimen voucher
150	*Chaetogaster langi* MOTU5	CH	Bern	Villeret	9	Suze River	Pascal Mulattieri	April 7, 2023	Régis Vivien	PP996453	PQ013429	PQ013517	GBIFCH 1222992
151	*Chaetogaster diastrophus* MOTU10	CH	Bern	Villeret	9	Suze River	Pascal Mulattieri	April 7, 2023	Régis Vivien	PP996454	PQ013430	PQ013518	GBIFCH 1222993
155	*Chaetogaster diastrophus* MOTU9	CH	Bern	Villeret	9	Suze River	Pascal Mulattieri	April 7, 2023	Régis Vivien	PP996455	PQ013431		
156	*Chaetogaster diastrophus* MOTU10	CH	Bern	Villeret	9	Suze River	Pascal Mulattieri	April 7, 2023	Régis Vivien	PP996456	PQ013432		GBIFCH 1222995
157	*Chaetogaster diastrophus* MOTU9	CH	Bern	Villeret	9	Suze River	Pascal Mulattieri	April 7, 2023	Régis Vivien	PP996457	PQ013433	PQ013519	GBIFCH 1222996
159	*Chaetogaster diastrophus* MOTU9	CH	Bern	Villeret	9	Suze River	Pascal Mulattieri	April 7, 2023	Régis Vivien	PP996458			GBIFCH 1222997
160	*Chaetogaster diastrophus* MOTU9	CH	Bern	Villeret	9	Suze River	Pascal Mulattieri	April 7, 2023	Régis Vivien	PP996459	PQ013434		GBIFCH 1222998
161	*Chaetogaster setosus* MOTU4	CH	Bern	Villeret	9	Suze River	Pascal Mulattieri	April 7, 2023	Régis Vivien	PP996460	PQ013435	PQ013520	GBIFCH 1222999
162	*Chaetogaster diastrophus* MOTU10	CH	Bern	Villeret	9	Suze River	Pascal Mulattieri	April 7, 2023	Régis Vivien	PP996461	PQ013436	PQ013521	GBIFCH 1223000
163	*Chaetogaster diastrophus* MOTU10	CH	Bern	Villeret	9	Suze River	Pascal Mulattieri	April 7, 2023	Régis Vivien	PP996462	PQ013437	PQ013522	GBIFCH 1223001
164	*Chaetogaster diastrophus* MOTU10	CH	Bern	Villeret	9	Suze River	Pascal Mulattieri	April 7, 2023	Régis Vivien	PP996463	PQ013438	PQ013523	GBIFCH 1223002
165	*Chaetogaster* sp. MOTU2	CH	Bern	Villeret	9	Suze River	Pascal Mulattieri	April 7, 2023	Régis Vivien	PP996464	PQ013439	PQ013524	GBIFCH 1223003
CDS1	*Chaetogaster diastrophus* MOTU3	CH	Aargau	Muri	Dws3	Bünz River	Régis Vivien et Benoit Ferrari	May 25 2021	Régis Vivien	PP996465	PQ013440	PQ013525	GBIFCH 1223004
CDS2	*Chaetogaster diastrophus* MOTU3	CH	Aargau	Muri	Dws3	Bünz River	Régis Vivien et Benoit Ferrari	May 25 2021	Régis Vivien	PP996466	PQ013441	PQ013526	GBIFCH 1223005
CDP1	*Chaetogaster diaphanus* MOTU6	CH	Aargau	Muri	Dws3	Bünz River	Régis Vivien et Benoit Ferrari	May 25 2021	Régis Vivien	PP996467			GBIFCH 1223006
CDP2	*Chaetogaster diaphanus* MOTU7	CH	Aargau	Muri	Dws3	Bünz River	Régis Vivien et Benoit Ferrari	May 25 2021	Régis Vivien	PP996468	PQ013442	PQ013527	GBIFCH 1223007
CDP3	*Chaetogaster diaphanus* MOTU6	CH	Aargau	Muri	Dws3	Bünz River	Régis Vivien et Benoit Ferrari	May 25 2021	Régis Vivien	PP996469	PQ013443	PQ013528	GBIFCH 1223008
CDP4	*Chaetogaster diaphanus* MOTU6	CH	Aargau	Muri	Dws3	Bünz River	Régis Vivien et Benoit Ferrari	May 25 2021	Régis Vivien	PP996470			GBIFCH 1223009

Isolate	Morphospecies / Group	Country	Canton	Locality	Site	Stream	Collected by	Collection date	Identified by	GenBank accession number COI	GenBank accession number ITS2	GenBank accession number 28S	Specimen voucher
CDP5	*Chaetogaster diaphanus* MOTU6	CH	Aargau	Muri	Dws3	Bünz River	Régis Vivien et Benoit Ferrari	May 25 2021	Régis Vivien	PP996471	PQ013444		GBIFCH 1223010
G4	*Chaetogaster diaphanus* MOTU6	CH	St. Gallen	Flawil	Dws2	Glatt River	Régis Vivien et Benoit Ferrari	May 31, 2022	Régis Vivien	PP996472	PQ013445		GBIFCH 1223011
Sor6	*Chaetogaster diaphanus* MOTU6	CH	Vaud	Ecublens	UNIL-Sorge	Sorge River	Régis Vivien	April 2020	Régis Vivien	PP996473	PQ013446	PQ013529	GBIFCH 1223012
Sor7	*Chaetogaster diaphanus* MOTU6	CH	Vaud	Ecublens	UNIL-Sorge	Sorge River	Régis Vivien	April 2020	Régis Vivien	PP996474	PQ013447	PQ013530	GBIFCH 1223013
Sor8	*Chaetogaster diaphanus* MOTU6	CH	Vaud	Ecublens	UNIL-Sorge	Sorge River	Régis Vivien	April 2020	Régis Vivien	PP996475	PQ013448	PQ013531	GBIFCH 1223014
Sor9	*Chaetogaster diaphanus* MOTU6	CH	Vaud	Ecublens	UNIL-Sorge	Sorge River	Régis Vivien	April 2020	Régis Vivien	PP996476	PQ013449	PQ013532	GBIFCH 1223015
Glatt6	*Chaetogaster* sp. MOTU8	CH	St. Gallen	Flawil	Dws4	Glatt River	Régis Vivien et Benoit Ferrari	October 28, 2020	Régis Vivien	PP996477	PQ013450	PQ013533	
183	*Chaetogaster diaphanus* MOTU7	CH	Aargau	Muri	Dws3	Bünz River	Régis Vivien et Benoit Ferrari	May 25 2021	Régis Vivien	PP996478	PQ013451	PQ013534	GBIFCH 1223017
184	*Chaetogaster diaphanus* MOTU7	CH	Aargau	Muri	Dws3	Bünz River	Régis Vivien et Benoit Ferrari	May 25 2021	Régis Vivien	PP996479	PQ013452	PQ013535	GBIFCH 1223018
185	*Chaetogaster diaphanus* MOTU7	CH	Aargau	Muri	Dws3	Bünz River	Régis Vivien et Benoit Ferrari	May 25 2021	Régis Vivien	PP996480	PQ013453		GBIFCH 1223019
186	*Chaetogaster diaphanus* MOTU7	CH	Aargau	Muri	Dws3	Bünz River	Régis Vivien et Benoit Ferrari	May 25 2021	Régis Vivien	PP996481	PQ013454	PQ013536	GBIFCH 1223020
187	*Chaetogaster diaphanus* MOTU7	CH	Aargau	Muri	Dws3	Bünz River	Régis Vivien et Benoit Ferrari	May 25 2021	Régis Vivien	PP996482	PQ013455	PQ013537	GBIFCH 1223021
189	*Chaetogaster diaphanus* MOTU7	CH	Aargau	Muri	Dws3	Bünz River	Régis Vivien et Benoit Ferrari	May 25 2021	Régis Vivien	PP996483	PQ013456	PQ013538	GBIFCH 1223022
190	*Chaetogaster diaphanus* MOTU7	CH	Aargau	Muri	Dws3	Bünz River	Régis Vivien et Benoit Ferrari	May 25 2021	Régis Vivien	PP996484	PQ013457	PQ013539	GBIFCH 1223023
191	*Chaetogaster diaphanus* MOTU7	CH	Aargau	Muri	Dws3	Bünz River	Régis Vivien et Benoit Ferrari	May 25 2021	Régis Vivien	PP996485	PQ013458	PQ013540	GBIFCH 1223024
192	*Chaetogaster diaphanus* MOTU7	CH	Aargau	Muri	Dws3	Bünz River	Régis Vivien et Benoit Ferrari	May 25 2021	Régis Vivien	PP996486	PQ013459	PQ013541	GBIFCH 1223025

Isolate	Morphospecies / Group	Country	Canton	Locality	Site	Stream	Collected by	Collection date	Identified by	GenBank accession number COI	GenBank accession number ITS2	GenBank accession number 28S	Specimen voucher
193	*Chaetogaster diaphanus* MOTU7	CH	Aargau	Muri	Dws3	Bünz River	Régis Vivien et Benoit Ferrari	May 25 2021	Régis Vivien	PP996487	PQ013460	PQ013542	GBIFCH 1223026
194	*Chaetogaster diaphanus* MOTU7	CH	Aargau	Muri	Dws3	Bünz River	Régis Vivien et Benoit Ferrari	May 25 2021	Régis Vivien	PP996488	PQ013461	PQ013543	GBIFCH 1223027
195	*Chaetogaster diastrophus* MOTU3	CH	Aargau	Muri	Dws3	Bünz River	Régis Vivien et Benoit Ferrari	May 25 2021	Régis Vivien	PP996489	PQ013462		GBIFCH 1223028
196	*Chaetogaster diastrophus* MOTU3	CH	Aargau	Muri	Dws3	Bünz River	Régis Vivien et Benoit Ferrari	May 25 2021	Régis Vivien	PP996490			GBIFCH 1223029
197	*Chaetogaster diastrophus* MOTU3	CH	Aargau	Muri	Dws3	Bünz River	Régis Vivien et Benoit Ferrari	May 25 2021	Régis Vivien	PP996491	PQ013463		GBIFCH 1223030
198	*Chaetogaster diastrophus* MOTU3	CH	Aargau	Muri	Dws3	Bünz River	Régis Vivien et Benoit Ferrari	May 25 2021	Régis Vivien	PP996492	PQ013464	PQ013544	GBIFCH 1223031
199	*Chaetogaster diastrophus* MOTU3	CH	Aargau	Muri	Dws3	Bünz River	Régis Vivien et Benoit Ferrari	May 25 2021	Régis Vivien	PP996493	PQ013465	PQ013545	GBIFCH 1223032
200	*Chaetogaster diastrophus* MOTU3	CH	Aargau	Muri	Dws3	Bünz River	Régis Vivien et Benoit Ferrari	May 25 2021	Régis Vivien	PP996494			GBIFCH 1223033
201	*Chaetogaster diaphanus* MOTU6	CH	Aargau	Muri	Dws3	Bünz River	Régis Vivien et Benoit Ferrari	May 25 2021	Régis Vivien	PP996495	PQ013466		GBIFCH 1223034
202	*Chaetogaster diaphanus* MOTU6	CH	Aargau	Muri	Dws3	Bünz River	Régis Vivien et Benoit Ferrari	May 25 2021	Régis Vivien	PP996496	PQ013467	PQ013546	GBIFCH 1223035
203	*Chaetogaster diaphanus* MOTU7	CH	Aargau	Muri	Dws3	Bünz River	Régis Vivien et Benoit Ferrari	May 25 2021	Régis Vivien	PP996497	PQ013468	PQ013547	GBIFCH 1223036
204	*Chaetogaster diaphanus* MOTU7	CH	Aargau	Muri	Dws3	Bünz River	Régis Vivien et Benoit Ferrari	May 25 2021	Régis Vivien	PP996498	PQ013469	PQ013548	
205	*Chaetogaster diaphanus* MOTU6	CH	Aargau	Muri	Dws3	Bünz River	Régis Vivien et Benoit Ferrari	May 25 2021	Régis Vivien	PP996499	PQ013470	PQ013549	
206	*Chaetogaster diaphanus* MOTU7	CH	Aargau	Muri	Dws3	Bünz River	Régis Vivien et Benoit Ferrari	May 25 2021	Régis Vivien	PP996500	PQ013471	PQ013550	GBIFCH 1223039

Isolate	Morphospecies / Group	Country	Canton	Locality	Site	Stream	Collected by	Collection date	Identified by	GenBank accession number COI	GenBank accession number ITS2	GenBank accession number 28S	Specimen voucher
207	*Chaetogaster diaphanus* MOTU7	CH	Aargau	Muri	Dws3	Bünz River	Régis Vivien et Benoit Ferrari	May 25 2021	Régis Vivien	PP996501	PQ013472	PQ013551	GBIFCH 1223040
209	*Chaetogaster diaphanus* MOTU6	CH	Aargau	Muri	Dws3	Bünz River	Régis Vivien et Benoit Ferrari	May 25 2021	Régis Vivien	PP996502	PQ013473	PQ013552	GBIFCH 1223041
210	*Chaetogaster diaphanus* MOTU7	CH	Aargau	Muri	Dws3	Bünz River	Régis Vivien et Benoit Ferrari	May 25 2021	Régis Vivien	PP996503	PQ013474	PQ013553	GBIFCH 1223042
211	*Chaetogaster diaphanus* MOTU7	CH	Aargau	Muri	Dws3	Bünz River	Régis Vivien et Benoit Ferrari	May 25 2021	Régis Vivien	PP996504	PQ013475	PQ013554	GBIFCH 1223043
212	*Chaetogaster diastrophus* MOTU3	CH	Aargau	Muri	Dws3	Bünz River	Régis Vivien et Benoit Ferrari	May 25 2021	Régis Vivien	PP996505	PQ013476		GBIFCH 1223044
213	*Chaetogaster diastrophus* MOTU3	CH	Aargau	Muri	Dws3	Bünz River	Régis Vivien et Benoit Ferrari	May 25 2021	Régis Vivien	PP996506	PQ013477	PQ013555	GBIFCH 1223045
214	*Chaetogaster diastrophus* MOTU3	CH	Aargau	Muri	Dws3	Bünz River	Régis Vivien et Benoit Ferrari	May 25 2021	Régis Vivien	PP996507			GBIFCH 1223046
215	*Chaetogaster diastrophus* MOTU3	CH	Aargau	Muri	Dws3	Bünz River	Régis Vivien et Benoit Ferrari	May 25 2021	Régis Vivien	PP996508	PQ013478	PQ013556	GBIFCH 1223047
216	*Chaetogaster diastrophus* MOTU3	CH	Aargau	Muri	Dws3	Bünz River	Régis Vivien et Benoit Ferrari	May 25 2021	Régis Vivien	PP996509	PQ013479		GBIFCH 1223048
219	*Chaetogaster diaphanus* MOTU7	CH	Aargau	Muri	Dws3	Bünz River	Régis Vivien et Benoit Ferrari	May 25 2021	Régis Vivien	PP996510	PQ013480	PQ013557	GBIFCH 1223049
220	*Chaetogaster diastrophus* MOTU10	CH	St. Gallen	Flawil	Dws4	Bünz River	Régis Vivien et Benoit Ferrari	October 28, 2020	Régis Vivien	PP996511	PQ013481		GBIFCH 1223050
221	*Chaetogaster diastrophus* MOTU10	CH	St. Gallen	Flawil	Dws4	Bünz River	Régis Vivien et Benoit Ferrari	October 28, 2020	Régis Vivien	PP996512	PQ013482		GBIFCH 1223051
222	*Chaetogaster diastrophus* MOTU10	CH	St. Gallen	Flawil	Dws4	Bünz River	Régis Vivien et Benoit Ferrari	October 28, 2020	Régis Vivien	PP996513	PQ013483		GBIFCH 1223052
223	*Chaetogaster diastrophus* MOTU10	CH	St. Gallen	Flawil	Dws4	Bünz River	Régis Vivien et Benoit Ferrari	October 28, 2020	Régis Vivien	PP996514	PQ013484		GBIFCH 1223053

Isolate	Morphospecies / Group	Country	Canton	Locality	Site	Stream	Collected by	Collection date	Identified by	GenBank accession number COI	GenBank accession number ITS2	GenBank accession number 28S	Specimen voucher
224	*Chaetogaster diastrophus* MOTU10	CH	St. Gallen	Flawil	Dws4	Bünz River	Régis Vivien et Benoit Ferrari	October 28, 2020	Régis Vivien	PP996515	PQ013485		GBIFCH 1223054
225	*Chaetogaster diastrophus* MOTU10	CH	St. Gallen	Flawil	Dws4	Bünz River	Régis Vivien et Benoit Ferrari	October 28, 2020	Régis Vivien	PP996516	PQ013486		GBIFCH 1223055
226	*Chaetogaster diastrophus* MOTU10	CH	St. Gallen	Flawil	Dws4	Bünz River	Régis Vivien et Benoit Ferrari	October 28, 2020	Régis Vivien	PP996517			GBIFCH 1223056
227	*Chaetogaster diastrophus* MOTU10	CH	St. Gallen	Flawil	Dws4	Bünz River	Régis Vivien et Benoit Ferrari	October 28, 2020	Régis Vivien	PP996518	PQ013487		GBIFCH 1223057
228	*Chaetogaster diastrophus* MOTU10	CH	St. Gallen	Flawil	Dws4	Bünz River	Régis Vivien et Benoit Ferrari	October 28, 2020	Régis Vivien	PP996519	PQ013488		GBIFCH 1223058
229	*Chaetogaster diastrophus* MOTU10	CH	St. Gallen	Flawil	Dws4	Bünz River	Régis Vivien et Benoit Ferrari	October 28, 2020	Régis Vivien	PP996520	PQ013489		GBIFCH 1223059
230	*Chaetogaster diastrophus* MOTU10	CH	St. Gallen	Flawil	Dws4	Bünz River	Régis Vivien et Benoit Ferrari	October 28, 2020	Régis Vivien	PP996521	PQ013490		GBIFCH 1223060
