# Supplementary figures and images for: The Genus Chaetogaster Baer, 1827 (Annelida, Clitellata) in Switzerland: A First Step toward Cataloguing Its Molecular Diversity and Description of New Species on a DNA Sequence Basis
Source: Biology (Basel). 2024 Sep 4;13(9):693. doi: 10.3390/biology13090693 (PMC11428632; doi:10.3390/biology13090693)

ASAP  
mPTP

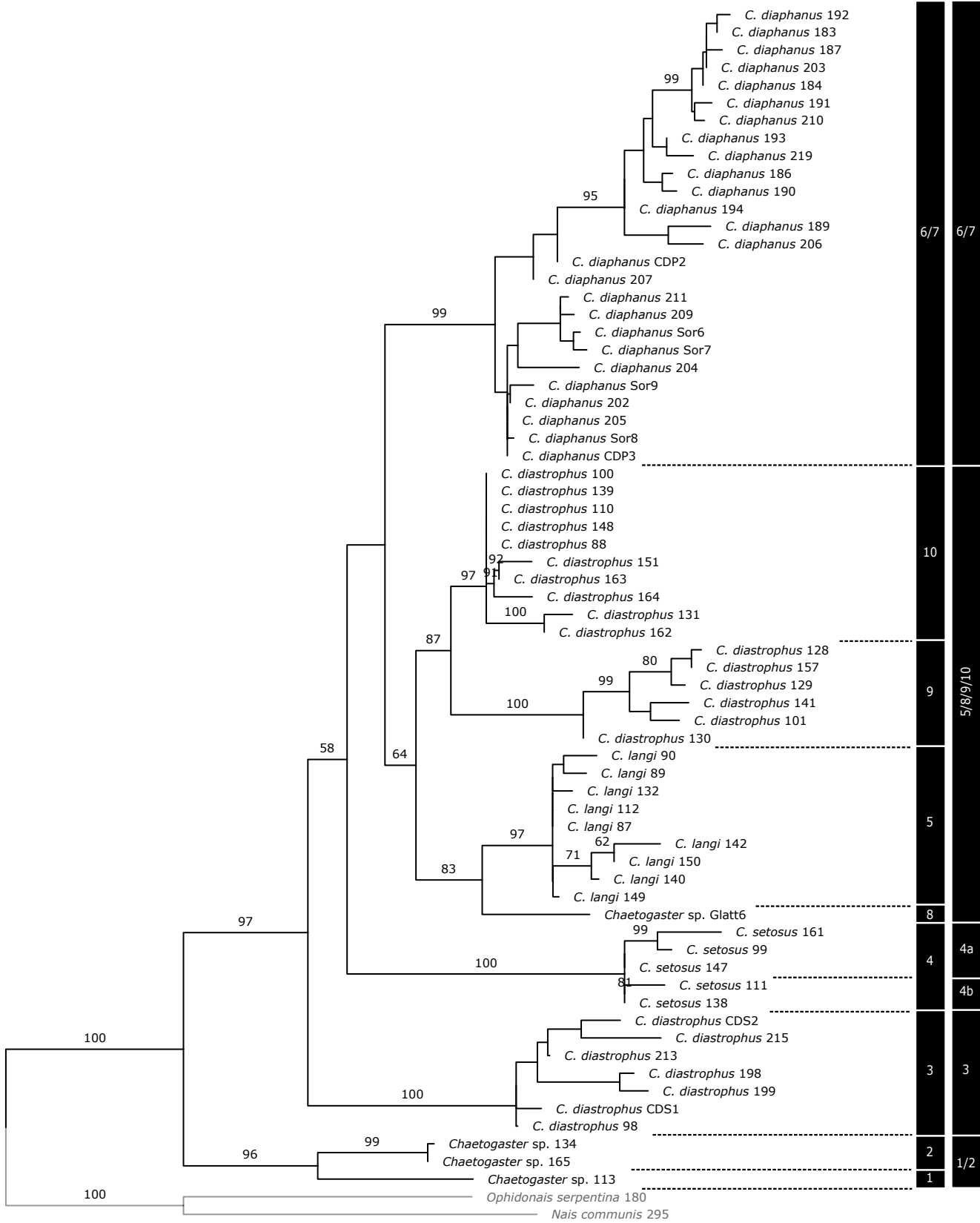

0.03

Supplement: Supplementary file 1 [file biology-13-00693-s001.zip › Figure_S1.pdf]
